# Influence of zirconium dioxide (ZrO_2_) and magnetite (Fe_3_O_4_) additions on the structural, electrical, and biological properties of Bioglass^®^ for metal implant coatings

**DOI:** 10.3389/fbioe.2025.1537856

**Published:** 2025-03-06

**Authors:** Imen Hammami, Manuel Pedro Fernandes Graça, Sílvia Rodrigues Gavinho, Joana Soares Regadas, Suresh Kumar Jakka, Ana Sofia Pádua, Jorge Carvalho Silva, Isabel Sá-Nogueira, João Paulo Borges

**Affiliations:** ^**1**^ I3N and Physics Department, Aveiro University, Aveiro, Portugal; ^2^ I3N-CENIMAT and Materials Science Department, NOVA School of Science and Technology, Campus de Caparica, Caparica, Portugal; ^3^ I3N-CENIMAT and Physics Department, NOVA School of Science and Technology, Campus de Caparica, Caparica, Portugal; ^4^ Associate Laboratory i4HB—Institute for Health and Bioeconomy, NOVA School of Science and Technology, NOVA University Lisbon, Caparica, Portugal; ^5^ UCIBIO—Applied Molecular Biosciences Unit, Department of Life Sciences, NOVA School of Science and Technology, NOVA University Lisbon, Caparica, Portugal

**Keywords:** bioglass^®^, zirconium, iron, bioactivity, antibacterial activity, electrical properties, bone regeneration, implant coatings

## Abstract

**Background:**

The growing need for durable implants, driven by aging populations and increased trauma cases, highlights challenges such as limited osseointegration and biofilm formation. 45S5 Bioglass^®^ has shown promise due to its bioactivity, antimicrobial properties, and ability to enhance osseointegration through electrical polarization. This study investigates the effects of incorporating different concentrations of ZrO_2_ and Fe_3_O_4_ into 45S5 Bioglass^®^ to enhance its electrical and biological properties.

**Methods:**

Raman analysis was used to evaluate how these oxides influenced the amount of non-bridging oxygens (NBOs) and glass network connectivity. Electrical characterization was performed using impedance spectroscopy to measure conductivity and ion mobility. Antibacterial activity was assessed using the agar diffusion method, and bioactivity was evaluated through simulated body fluid (SBF) immersion tests.

**Results:**

The results revealed that bioglasses containing ZrO_2_ exhibited higher NBO content compared to Fe_3_O_4_, leading to improved electrical and biological properties. ZrO_2_, particularly at 2 mol%, significantly enhanced conductivity, antibacterial activity, and bioactivity. In contrast, Fe_3_O_4_ reduced both antibacterial activity and bioactivity.

**Conclusion:**

The findings demonstrate that ZrO_2_ addition improves the electrical and biological performance of 45S5 Bioglass^®^, making it a promising candidate for durable implants. Fe_3_O_4_, however, showed limited benefits.

## 1 Introduction

Dental implants are fundamental to contemporary restorative dentistry, providing an exceptional solution for individuals experiencing tooth loss due to aging, trauma, or disease. The global demand for dental implants has increased significantly, driven by an aging population, increased awareness of oral health, and advancements in implant technology. These implants are essential for restoring oral function, aesthetics, and overall quality of life, making them a primary focus in biomedical research (Addy, 2024; Cociuban et al., 2024). Despite significant progress, the long-term efficacy of dental implants remains uncertain. Osseointegration, the biological process through which the implant integrates with surrounding bone tissue, is crucial for stability and functionality. Titanium and its alloys, particularly Ti-6Al-4V, are widely used for their superior mechanical properties and corrosion resistance. However, they may have limitations in facilitating osseointegration (Civantos et al., 2017; Silva et al., 2022). Insufficient bone integration can lead to micromotion and gaps at the implant-bone interface, which may promote bacterial adhesion and biofilm formation. Once established, these biofilms can induce persistent infections and localized bone resorption, ultimately threatening implant success (Gbejuade et al., 2015; Davidson et al., 2019).

To address these limitations, researchers are exploring bioactive materials and coatings promoting osseointegration while reducing bacterial colonization risk. One of the most promising materials is 45S5 Bioglass^®^ (46.1SiO_2_-24.4Na_2_O-26.9CaO-2.6P_2_O_5_ (mol%)), which gained significant attention for its ability to promote bone regeneration (Hench et al., 1971; Hench and Paschall, 1973). Initially developed in the 1970s by Larry L. Hench (Hench, 2006; Hench, 2013), these glasses possess a unique composition that allows them to bond directly to living bone tissue. Upon contact with physiological fluids, 45S5 Bioglass^®^ undergoes a series of reactions, leading to the formation of a hydroxyapatite layer on its surface. This layer mimics the mineral component of natural bone, facilitating osseointegration. Additionally, the release of ions from the glass can inhibit bacterial growth, reducing the risk of infection (Allan et al., 2001; Allan et al., 2002; Begum et al., 2016).

The incorporation of metal ions into bioglass has emerged as a promising strategy to enhance its biological properties (Cacciotti, 2017; de Souza Balbinot et al., 2019; Malavasi et al., 2019; Aghili et al., 2022; Hammami et al., 2024). Elements such as zirconium (Zr) and iron (Fe) have garnered significant attention due to their unique characteristics. Zirconium, particularly in the form of zirconium dioxide (ZrO_2_), is extensively utilized in biomedical applications due to its biocompatibility and outstanding mechanical properties, including its exceptional strength and fracture toughness, making it a widely used reinforcing agent (Silva et al., 2004; Pattnaik et al., 2011; Bhowmick et al., 2017; Kang et al., 2021). Zr can also stimulate osteoblast proliferation and differentiation, leading to accelerated bone healing (Hempel et al., 2010; Pattnaik et al., 2011; Bhowmick et al., 2017). Studies by Goo et al. (2018) and Sa et al. (2018) have demonstrated the effectiveness of ZrO_2_ in promoting bone formation and improving osteogenic activity, respectively. Additionally, ZrO_2_ possesses significant antimicrobial properties against various bacteria by interfering with bacterial respiration processes (Jangra et al., 2012; Fathima et al., 2017; Rad Goudarzi et al., 2019; Kumar et al., 2020).

Iron (Fe) is essential for various cellular functions, including oxygen transport and energy metabolism (Touati, 2000; Theil and Goss, 2009). Iron deficiency can lead to impaired collagen synthesis and reduced bone density (Abraham, 2014; Bose et al., 2018). Fe supports osteoblastic differentiation, proliferation, and calcification (Ullah et al., 2020; Zhu et al., 2023). Research by Long et al. (2014) and Zhou et al. (2024) has shown that incorporating Fe into biomaterials enhances cell adhesion, proliferation, and osteogenic differentiation. Fe also exhibits antibacterial properties by generating reactive oxygen species (ROS) through the Fenton reaction, which can damage bacterial cells (Touati, 2000; Behera et al., 2012; Zhang et al., 2016; Ezealigo et al., 2021).

In addition to ion insertion, electrical polarization offers a promising approach to enhance the biological properties of bioactive glass. By applying an electric field, surface charges can be induced, influencing cellular interactions and promoting tissue integration (Metwally and Stachewicz, 2019). This approach has been successfully applied to calcium-phosphate ceramics like hydroxyapatite (HA), where negative surface charges have been shown to promote bone growth and cell proliferation (Yamashita et al., 1996; Kobayashi et al., 2001; Ohgaki et al., 2001). However, the application of electrical polarization to bioglasses remains relatively unexplored. While the electrical polarization of HA is primarily driven by proton migration (Prezas et al., 2017), the higher ionic conductivity of bioglasses, mainly due to sodium ions, suggests that ion migration may play a significant role in their polarization (Obata et al., 2003; Obata et al., 2004). A deeper understanding of the electrical properties of bioglass is crucial to unlock their potential for electrical polarization, which can significantly enhance their bioactivity and overall performance in biomedical applications.

This study addresses a significant challenge in dental implantology: bacterial infections that can lead to bone loss and subsequent implant failure. To mitigate this issue, we developed a material for implant coating based on 45S5 bioglass^®^, incorporating varying concentrations of zirconium dioxide (ZrO_2_) and magnetite (Fe_3_O_4_). The melt-quenching technique used in this study offers significant advantages in terms of scaling up the production of these materials for clinical use. This method allows for the fabrication of bioactive glasses in large quantities at a relatively low cost compared to other techniques, such as sol-gel. Its reliability and efficiency make it a promising approach for large-scale production, which is essential for practical applications in clinical settings. The influence of these metal oxides on the structural properties of 45S5 bioglass was examined using X-ray diffraction (XRD), Fourier-transform infrared (FTIR), and Raman spectroscopy. Given bioglass’s potential for electrical charge storage, impedance spectroscopy (IS) was employed to investigate its electrical properties and the impact of oxide additions. To evaluate the potential of the prepared glasses as implant coating materials, a cytotoxicity assay was conducted using the extract method and human osteosarcoma (Saos-2) cells. The antibacterial activity of the different bioglass compositions was assessed using the agar diffusion method against *Escherichia coli*, *Staphylococcus aureus*, and *Streptococcus mutans*. The bioactivity assay was assessed by immersing the samples in a Simulated Body Fluid (SBF) solution.

## 2 Materials and methods

### 2.1 Bioglass synthesis

Bioglass samples were fabricated using the melt-quenching technique, adhering to the 45S5 Bioglass^®^ composition (46.1SiO_2_-24.4Na_2_O-26.9CaO-2.6P_2_O_5_ (mol%)) as described by Hench et al. (1971), Hench and Paschall (1973). Various concentrations (1, 2, and 4 mol%) of ZO_2_ (Zr1, Zr2, Zr4) and Fe_3_O_4_ (Fe1, Fe2, Fe4) were incorporated into the bioglass network. High-purity (≥99%) chemical precursors (SiO_2_, P_2_O_5_, CaCO_3_, Na_2_CO_3_, and (ZrO(NO_3_)_2_∙XH_2_O; X∼3) or Fe_3_O_4_), supplied by sigma-Aldrich, were mixed using a planetary ball mill system for 1 h at 300 rpm. The resulting mixture was calcined at 800 °C for 8 h, followed by melting at 1,400 °C for 1 h. To enhance homogeneity, the melt was re-melted under identical conditions. The resulting bulk glass was ground and milled using planetary ball milling process, to obtain the final bioglass powders. These synthesis parameters were consistently applied to all the compositions, including the varying concentrations of ZrO_2_ (1, 2, and 4 mol%) and Fe_3_O_4_ (1, 2, and 4 mol%). The selected parameters were verified to ensure reproducibility, with the combination of visual inspection and re-melting steps confirming the suitability of the process for producing homogeneous bioglass samples. The nominal compositions of the prepared bioglass samples are presented in Table 1.

**TABLE 1:** Composition of different bioglass samples.

Composition (mol%)						
Sample	SiO_2_	Na_2_O	CaO	P_2_O_5_	ZrO_2_	Fe_3_O_4_
BG	46.10	24.40	26.90	2.60	-	-
Zr1	45.64	24.16	26.63	2.57	1	-
Zr2	45.18	23.91	26.36	2.55	2	-
Zr4	44.26	23.42	25.82	2.50	4	-
Fe1	45.64	24.16	26.63	2.57	-	1
Fe2	45.18	23.91	26.36	2.55	-	2
Fe4	44.26	23.42	25.82	2.50	-	4

### 2.2 Structural characterization

X-ray diffraction (XRD) analysis was performed using a Panalytical Aeris diffractometer with Cu Kα radiation (λ = 1.54056 Å). Data were collected over a 2θ range of 10°–70° with a step size of 0.002°.

Fourier Transform Infrared (FTIR) spectroscopic analysis was performed using a PerkinElmer Spectrum BX spectrometer equipped with a Golden Gate Diamond Attenuated Total Reflectance (ATR) accessory. Powdered samples were analysed at room temperature and humidity (approximately 23°C and 35%) over a spectral range of 400–1,200 cm^−1^.

Raman spectroscopic analysis was conducted on the bulk samples using a Jobin Yvon HR800 spectrometer. An Ar^+^ laser (λ = 532 nm) was employed, and spectra were acquired in backscattering geometry over the spectral range of 200–1,400 cm^−1^.

### 2.3 Electrical properties

Electrical measurements were performed on 1 mm-thick bulk glass samples. Silver conducting paste was applied to the opposing parallel surfaces of the samples to establish good electrical contact. Both direct current (DC) and alternating current (AC) measurements were conducted in a nitrogen bath cryostat, enabling precise temperature control within a range of 100–400 K. An Oxford Research IT-C4 system equipped with a platinum sensor was used to monitor and control the sample temperature. DC conductivity measurements were performed using a Keithley 617A electrometer, applying a constant voltage of 100 V across the bulk glass sample. AC impedance spectroscopy measurements were carried out using an Agilent 4294A impedance analyser over a frequency range of 100 Hz to 1 MHz, employing the C_p_−R_p_ configuration and applying an ac signal of 0.5 V. The complex permittivity (ε^*^) and complex modulus (M^*^) were calculated using the following Equations (Graça et al., 2012; Ashok et al., 2017; Barsoukov and Macdonald, 2018).

$$\varepsilon^{*}=\varepsilon^{\prime }-j\varepsilon^{\prime \prime }=C_{p}\;\frac{d}{\varepsilon_{0}A}-i\;d\;\left({\omega R_{P}\varepsilon }_{0}A\right)$$

$$M^{*}=\frac{1}{\varepsilon^{*}}=M^{\prime }+iM^{\prime \prime }=\frac{\varepsilon^{\prime }}{\varepsilon^{\prime 2}+\varepsilon^{\prime \prime 2}}+i\;\frac{\varepsilon^{\prime \prime }}{\varepsilon^{\prime 2}+\varepsilon^{\prime \prime 2}}$$

where C_p_ and R_p_ are the measured capacitance and resistance, d is the sample thickness, A is the electrode area, ω is the angular frequency, and ε_0_ is the permittivity of the free space (8.8542 × 10^−12^ F/m).

The complex ac conductivity (σ_ac_) was calculated using the following relation (El-Mallawany, 2014; Barsoukov and Macdonald, 2018):

$${\sigma_{ac}}^{*}=\varepsilon_{0}\omega \varepsilon^{\prime \prime }+i\varepsilon_{0}\omega \varepsilon^{\prime }$$

The activation energy (Ea) for both AC and DC conductivity was determined by fitting the temperature-dependent conductivity data to the Arrhenius equation (Macdonald, 1987; Barsoukov and Macdonald, 2018).

$$\sigma =\sigma_{0}\exp\left(\frac{-E_{a}}{k_{B}T}\right)$$

where σ_0_ is a pre-exponential factor, E_A_ is the activation energy, K_B_ is the Boltzmann constant, and T is the temperature.

### 2.4 Cytotoxicity assay

The cytotoxicity of the bioglass powders was evaluated using a standard extract test with human osteosarcoma (Saos-2) cells (ATCC^®^ HTB-85™), following the International Standard ISO 10993-5. Samples were sterilized at 120 °C for 2 h prior to testing. Non-passivated extracts were prepared by incubating the powders in McCoy 5A medium at a concentration of 100 mg/mL for 24 h at 37°C, followed by filtration. For the passivated extract, the filtered powders were re-incubated in fresh McCoy 5A medium for 24 h. Saos-2 cells were seeded in 96-well plates at a density of 30,000 cells/cm^2^, incubated for 24 h at 37°C in a 5% CO_2_ atmosphere, and then exposed to the diluted extracts (50%, 25%, 12.5%, and 6.25%). A positive control, consisting of cells exposed to a cytotoxic environment induced by 10% dimethyl sulfoxide (DMSO), and a negative control of viable cells were included. After 48 h, Cytotoxicity was evaluated after 48 h of cell incubation with the extracts and their dilutions using a resazurin-based colorimetric assay. Cell viability was assessed by measuring the absorbance of each well at 570 nm and 600 nm using a BioTek ELx800UV microplate reader. Each experiment was performed in triplicate, each comprising six technical replicates.

### 2.5 Antibacterial activity

The antibacterial properties of the bioglass compositions were assessed using an agar diffusion assay. Reference strains of *E.scherichia coli* K12, *Staphylococcus aureus* COL MRSA (methicillin-resistant), and *Streptococcus mutans* DSM20523 were cultured overnight in tryptic soy broth (TSB) at 37°C. Tryptic Soy Broth (TSB) medium solidified with Tryptic Soy Agar (TSA) was used to prepare two-layer bioassay plates. The base layer was 1.5% w/v and the top layer was 0.8% w/v. The top layer was inoculated with approximately 10^8^ CFU/mL of the appropriate indicator bacteria. Sterilized cylindrical bioglass pellets (6 mm diameter, ∼2 mm thick) were placed on agar plates, and incubated for 24 h at 37°C. *S. mutans* plates were incubated in a 5% CO_2_ atmosphere. Zones of inhibition were measured to determine the antibacterial efficacy of the bioglass compositions. To analyse the statistical significance of the results, an unpaired t-test was performed on the data from eight independent assays for each bacterial strain. Statistical comparisons between the base bioglass composition and the modified compositions were conducted using GraphPad Prism 8.0 software.

### 2.6 Bioactivity assay

Bioactivity assessments were performed following the International standard ISO 23317:2017. Bioactive glass pellets with 7 mm in diameter were immersed in simulated body fluid (SBF) and incubated at 37°C with orbital shaking for 12, 24, 48, 96 h, 14, and 28 d. SBF solution was refreshed every 48 h to simulate physiological conditions. The SBF volume (V_S_) for each pellet was calculated using:

$$V_{s}=100\;mm\times S_{a}$$

Where V_S_ represents the SBF volume in mm^3^, and S_A_ denotes the pellet’s surface area in mm^2^.

Following incubation, the pellets were extracted from the SBF, gently rinsed with deionized water, and subsequently dried at room temperature. The morphological and compositional alterations induced by the reaction with SBF were examined using scanning electron microscopy-energy dispersive X-ray spectroscopy (SEM-EDS).

## 3 Results and discussion

### 3.1 Structural characterization

**FIGURE 1:**
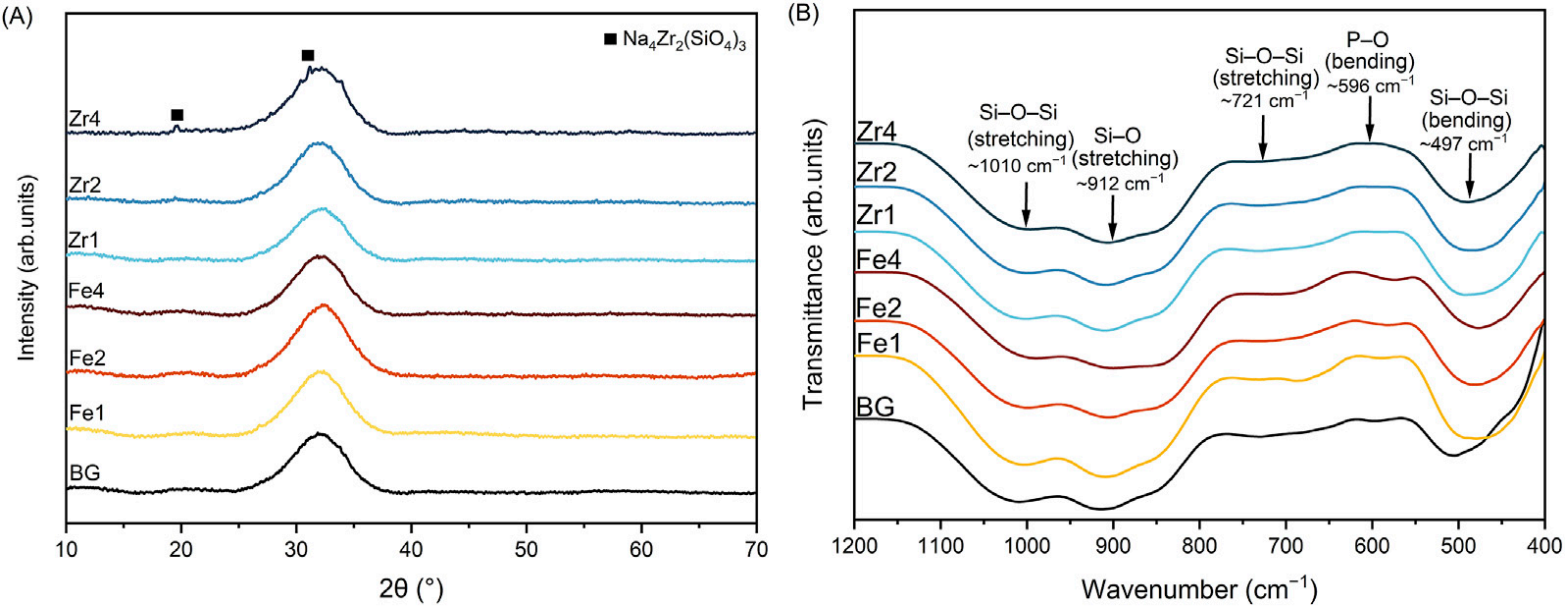
**(A)** XRD patterns, and **(B)** FTIR spectra of the bioglass samples.

Figure 1A presents XRD patterns of all synthesized bioglass samples. The 45S5 Bioglass (referred to as BG) displayed a broad diffraction peak between 25° and 38°, characteristic of amorphous materials. This indicates the absence of long-range atomic order within the glass structure (Lopes et al., 2014; Araujo et al., 2020). The addition of Fe_3_O_4_ did not alter the bioglass structure. Similarly, a small content of ZrO_2_ (2 mol%) maintained the glass’s amorphous nature. However, increasing ZrO_2_ concentration led to the emergence of a crystalline phase identified as sodium zirconium silicate (Na_4_Zr_2_(SiO_4_)_3_) with a hexagonal crystal structure.

Figure 1B depicts the FTIR spectra of the prepared bioglass samples. The BG exhibits prominent absorption bands at approximately 1,010, 912, 721, 596, and 497 cm^−1^. The bands at 1,010 cm^−1^ and 721 cm^−1^ correspond to Si-O-Si stretching vibrations (Boccaccini et al., 2007; Ibrahim et al., 2017; Ibrahim et al., 2018). The presence of a band at 912 cm^−1^ indicates the existence of non-bridging oxygen ions (NBOs), associated with Si-O-NBO stretching (Boccaccini et al., 2007; Ibrahim et al., 2017; Ibrahim et al., 2018). The band at 596 cm^−1^ is attributed to P-O bending vibrations in amorphous phosphate, while the band at 497 cm^−1^ corresponds to Si-O-Si bending vibrations (Boccaccini et al., 2007; Dziadek et al., 2016; El-Rashidy et al., 2017; Ibrahim et al., 2017; Ibrahim et al., 2018). The incorporation of Fe_3_O_4_ and ZrO_2_ does not significantly alter the FTIR spectra of the bioglass.

**FIGURE 2:**
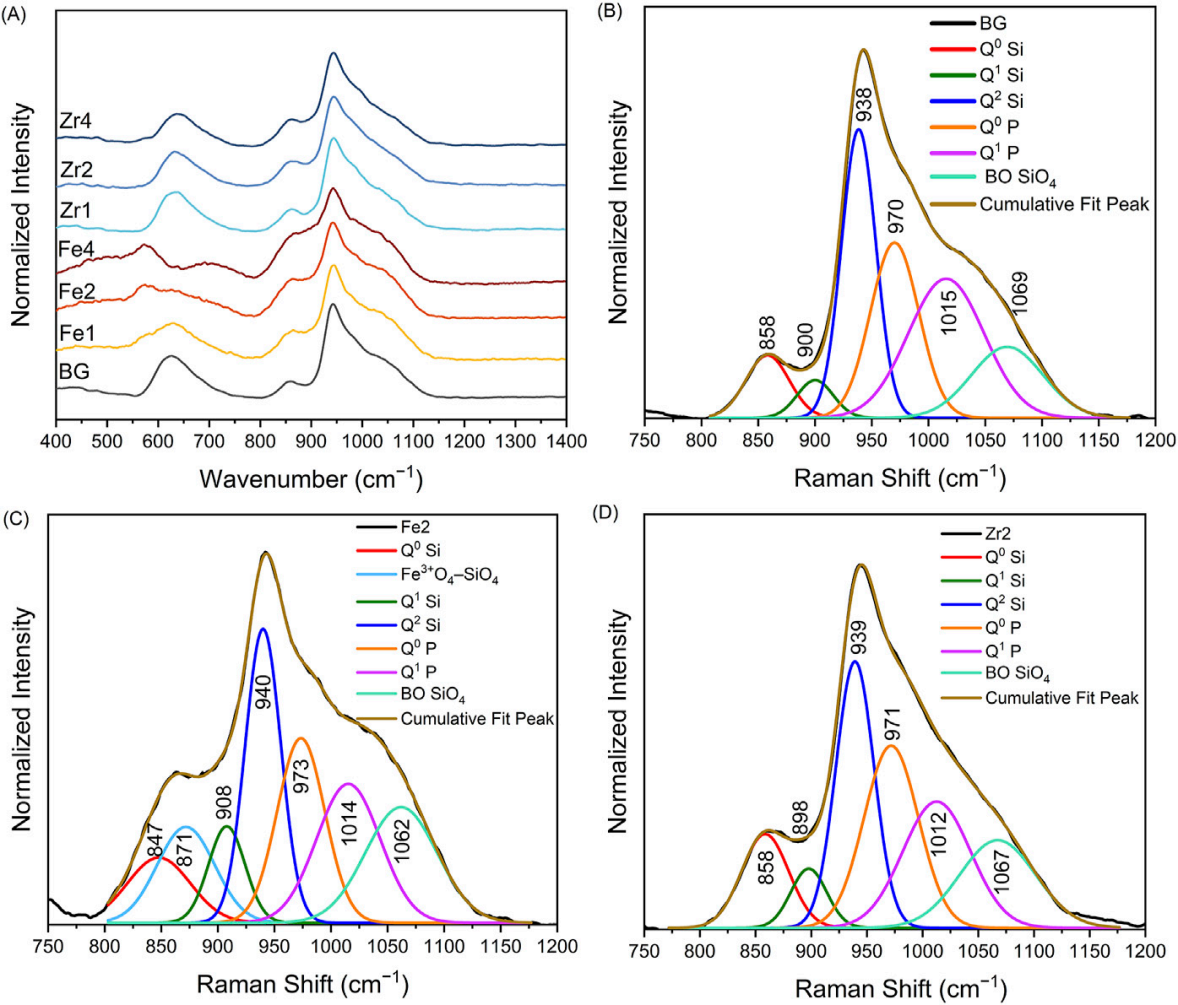
**(A)** Raman spectra of different bioglasses; deconvoluted Raman spectra of **(B)** BG, **(C)** Fe2, and **(D)** Zr2 bioglasses.

The Raman spectra of all bioglass compositions are shown in Figure 2A. The BG spectrum can be divided into two regions: low-wavenumber (<750 cm^−1^) and high-wavenumber (>750 cm^−1^). The broad band at 630 cm^−1^ in the low-wavenumber region is attributed to the rocking motion of bridging oxygen (BO) in structural units containing non-bridging oxygen (NBO) and symmetric Si-O-Si bending of three-membered rings (Aguiar et al., 2008; Aguiar et al., 2009; Lopes et al., 2014). In the high-wavenumber region, the deconvoluted Raman spectrum (Figure 2B) reveals six vibrational modes at approximately 858, 900, 938, 970, 1,015, and 1,069 cm^−1^. These modes correspond to the symmetric stretching of Q^0^ Si, Q^1^ Si, Q^2^ Si, Q^0^ P, Q^1^ P units, and BO in all Q Si species, respectively (Aguiar et al., 2009; Dziadek et al., 2016; Araujo et al., 2020). The addition of Fe_3_O_4_ modifies the glass structure, as evidenced by the Raman spectra in Figure 2C. As the Fe_3_O_4_ content rises, the intensity of the band at 630 cm^−1^ decreases, and new bands emerge. The appearance of a band at approximately 730 cm^−1^ is associated with the formation of Fe-related structural units, as reported in the literature for nano-Fe_3_O_4_ and Fe_3_O_4_-doped silicate glasses (Li et al., 2012; Nayak and Desa, 2018). Additionally, the emergence of bands at 560 cm^−1^ and 480 cm^−1^, which intensify with increasing Fe_3_O_4_ content, correlates with the vibrational modes of hematite (Chamritski and Burns, 2005; Zhang et al., 2022). In the high-wavenumber region, the deconvolution of the Raman spectra in Figure 4. 8 reveals a new band around 880 cm^−1^ for Fe_3_O_4_-modified glasses, with its intensity increasing with Fe_3_O_4_ content. This band is associated with vibrations involving Fe^3+^-O-Si bridging oxygen atoms or a coupled Fe^3+^O_4_-SiO_4_ mode (Wang et al., 1995; Di Muro et al., 2009; Baert et al., 2011). The formation of these new bonds suggests increased rigidity within the glass network. The addition of ZrO_2_ does not substantially modify the Raman spectra of the bioglass. Figure 2D illustrates the deconvoluted Raman spectra for the Zr2 samples, emphasizing the changes in the intensity of vibrational bands associated with NBOs.

**FIGURE 3:**
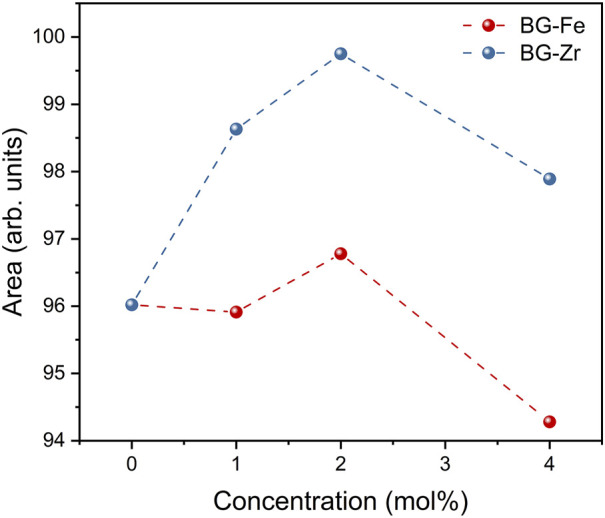
The sum of the areas of the Raman vibration bands associated with NBOs.

Figure 3 shows the sum of the area of Raman vibration bands associated with NBOs (Q^0^, Q^1^, Q^2^, and Q^3^ units) for the bioglasses modified with ZrO_2_ and Fe_3_O_4_. The incorporation of up to 2 mol% ZrO_2_ leads to an increase in NBO concentration. Conversely, further ZrO_2_ addition to 4 mol% results in a decline in NBOs, likely due to the formation of crystalline phases, as evidenced by XRD data, which increases the glass network’s connectivity. For bioglasses modified with Fe_3_O_4_, a slight NBO increase was observed in the glass with 2mol% Fe_3_O_4_ compared to the 45S5 bioglass, followed by a decrease with higher Fe_3_O_4_ content. This can be attributed to the emergence of the coupled Fe^3+^O_4_–SiO_4_ mode, (Figure 2C), suggesting that Fe forms new bonds with silica tetrahedra, thereby increasing the glass network’s rigidity.

### 3.2 Electrical properties

**FIGURE 4:**
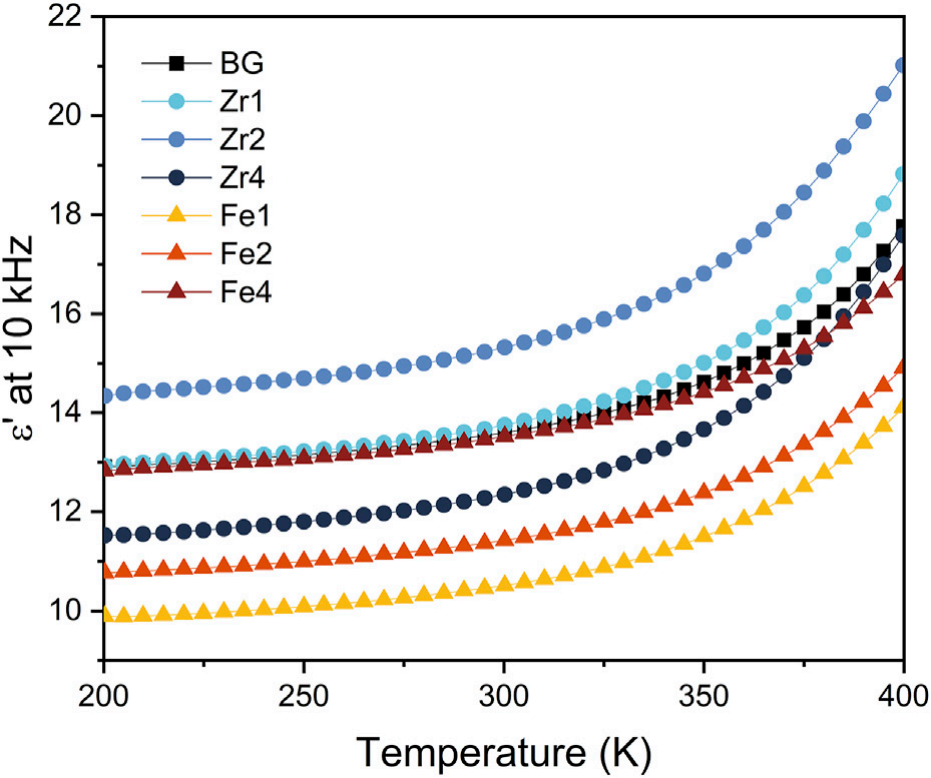
The variation dielectric constant, ε′, as a function of the temperature for all bioglass samples.

Figure 4 shows the variation of the dielectric permittivity (ε′) with temperature at a fixed frequency of 10 kHz. The dielectric constant increases with rising temperature in all samples which can be ascribed to the increased mobility of dipoles within the glass network at higher temperatures. This thermally activated dipole reorientation enhances the material’s polarizability under the applied electric field, leading to higher dielectric constant values. Based on Figure 4 and Table 2, the insertion of ZrO_2_ and Fe_3_O_4_ into the glasses network affects the dielectric constant values. A decrease in dielectric constant was observed for all glasses modified with Fe_3_O_4_ compared to the 45S5 bioglass. Furthermore, an increase in the dielectric constant was noted as the Fe_3_O_4_ concentration in the bioglass network increased. This increase can be attributed to the structural modifications introduced by the Fe_3_O_4_ addition. Raman spectroscopy revealed the formation of Fe^3+^-O-Si bonds and Fe-related structural units. These bonds enhance the polarizability of the glass network due to the high ionic polarizability of Fe^3+^ ions and the localized dipoles formed within the structure. Furthermore, the incorporation of 4 mol% Fe_3_O_4_ reduces the concentration of non-bridging oxygen ions (NBOs), leading to increased network connectivity (Figure 3). Although the glass rigidity at high Fe_3_O_4_ content, the greater polarizability introduced by Fe-related units dominates, resulting in an overall enhancement of the dielectric constant. In contrast, the ZrO_2_-modified glasses exhibit a different trend. While initial additions of ZrO_2_ (up to 2 mol%) lead to a slight increase in the dielectric constant due to increased NBOs, further increases in the concentration of ZrO_2_ to 4 mol% result in a decrease in the dielectric constant. This decrease could be related to the presence of the Na_4_Zr_2_(SiO_4_)_3_ crystalline phase, which increases the rigidity of the bioglass, leading to a reduction in NBOs and, consequently, a lower dielectric constant.

**TABLE 2:** The dielectric constant (ε′), dielectric loss (tan δ), AC, conductivity (σ_AC_), AC, activation energy E_a_ (AC), DC conductivity (σ_DC_), and DC activation energy E_a_ (DC) for all BG samples.

Sample	ε′	tan δ (10^–2^)	σ_ac_ (10^–7^) [S/m]	E_a_ (AC) [kJ/mol]	σ_dc_ (10^–9^) [S/m]	E_a_ (DC) [kJ/mol]
	(300 K; 10 kHz)			(10 kHz)	(300 K)	
BG	13.59 ± 0.72	1.58 ± 0.02	1.19 ± 0.01	37.95 ± 0.98	0.91 ± 0.08	75.82 ± 0.79
Zr1	13.75 ± 1.92	2.02 ± 0.01	1.54 ± 0.07	39.09 ± 0.92	1.61 ± 0.16	73.20 ± 0.76
Zr2	15.32 ± 1.95	2.28 ± 0.03	1.94 ± 0.04	37.90 ± 0.78	1.19 ± 0.17	75.96 ± 0.79
Zr4	12.34 ± 1.53	2.37 ± 0.01	1.62 ± 0.09	38.68 ± 0.87	1.45 ± 0.19	73.20 ± 0.76
Fe1	10.51 ± 1.02	1.93 ± 0.03	1.13 ± 0.02	36.97 ± 0.64	0.25 ± 0.04	80.52 ± 0.62
Fe2	11.42 ± 1.68	1.7 ± 0.02	1.14 ± 0.06	36.55 ± 0.69	0.71 ± 0.07	75.65 ± 0.44
Fe4	13.52 ± 1.23	1.45 ± 0.02	1.09 ± 0.03	35.20 ± 0.75	0.09 ± 0.002	85.94 ± 0.89

**FIGURE 5:**
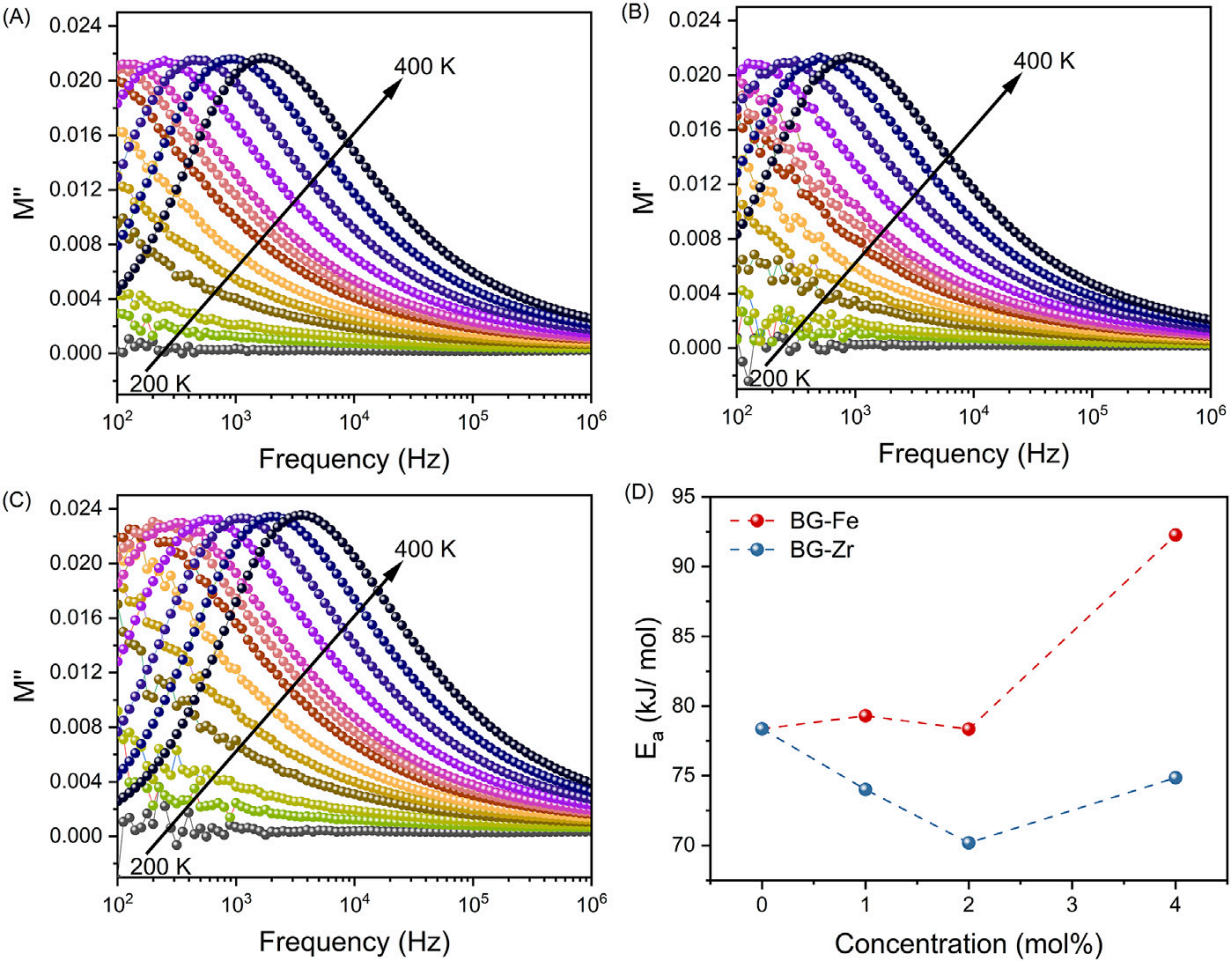
The imaginary part of the electrical modulus M″ versus frequency for **(A)** BG, **(B)** Fe4, and **(C)** Zr4 samples; **(D)** The variation of the activation energy E_a_ with increasing ZrO_2_ and Fe_3_O_4_ concentration.

The dielectric properties of the bioglasses were investigated using the modulus formalism (M*). This approach mitigates the influence of low-frequency effects, such as electrode polarization and conductivity, providing a clearer view of the dielectric relaxation processes (Silva et al., 2005). As shown in Figure 5, a single relaxation peak is observed, which shifts to higher frequencies with increasing temperature, indicating a thermally activated mechanism. This relaxation behaviour, more evident in the modulus representation than in other representations like permittivity or impedance, is attributed to the formation of dipoles involving network modifier ions and NBOs. The activation energy (E_a_) associated with the dielectric relaxation process was determined from the temperature dependence of the relaxation frequency, obtained from the imaginary part of the electrical modulus (M″), and analysing the data with the Arrhenius model (Figure 5D). Adding a low concentration of ZrO_2_ (up to 2 mol%) to the bioglass network decreases the activation energy. This can be attributed to the network-modifying role of ZrO_2_, which breaks down the silica network and introduces NBO sites. This increased disorder facilitates ion movement, lowering the activation energy. As ZrO_2_ concentration rises, its role shifts towards network stabilization, leading to a decrease in NBOs and increased structural integrity. The emergence of crystalline phases, as observed in the XRD patterns, further supports this network polymerization. Consequently, the activation energy increases as ions and dipoles require more energy to relax within the more rigid network. Regarding the effect of Fe_3_O_4_, the addition of low concentrations (up to 2 mol%) does not significantly affect the activation energy. However, at higher Fe_3_O_4_ concentrations, the activation energy increases, suggesting increased network rigidity. This is attributed to the formation of new cross-linking bonds between Fe ions and silica tetrahedra, as evidenced by Raman spectroscopy. The increased network connectivity and reduced NBOs hinder ion mobility, leading to higher activation energies. Moreover, it is noted that the activation energy for Fe_3_O_4_-modified bioglasses is higher than that of ZrO_2_-modified bioglasses due to the stronger cross-linking effect of Fe^3+^-O-Si bonds. The weaker connectivity in bioglasses with ZrO_2_ compared to those with Fe_3_O_4_, as evidenced in Figure 3 by the higher NBOs in ZrO_2_-modified bioglasses, results in lower E_a_. This highlights the greater impact of Fe_3_O_4_ on network reinforcement.

**FIGURE 6:**
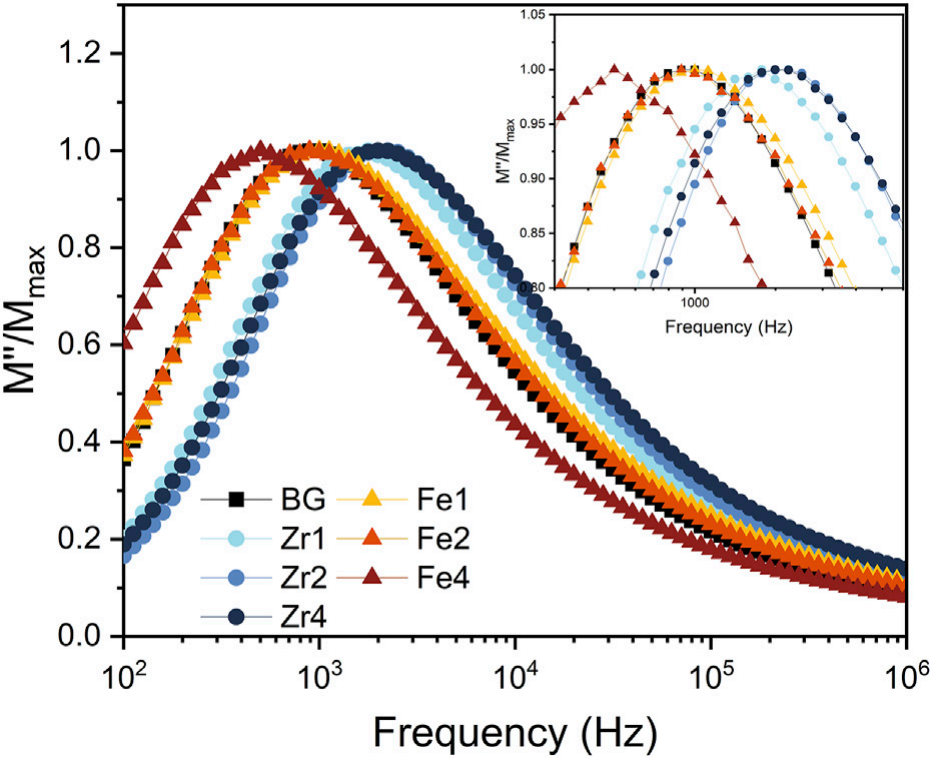
The normalized imaginary part of the modulus M″/M″_max_ versus the frequency at 390 K for all bioglasses.

Figure 6 depicts the normalized imaginary part of the electric modulus (M″/M″_max_) versus frequency for all bioglass samples. It can be seen that the insertion of ZrO_2_ into the glass network shifts the relaxation peak to higher frequencies, corresponding to a decrease in relaxation time. In contrast, the addition of Fe_3_O_4_ up to 2 mol% does not significantly affect the relaxation behaviour. However, further increasing the Fe_3_O_4_ content to 4 mol% shifts the relaxation peak to lower frequencies, suggesting an increase in relaxation time. This suggests that excess Fe_3_O_4_ restricts the alignment of dipoles within the glass network in response to an applied electric field. The observed variations in relaxation behaviour are attributed to structural modifications within the glass, specifically related to alterations in the NBOs content.

**FIGURE 7:**
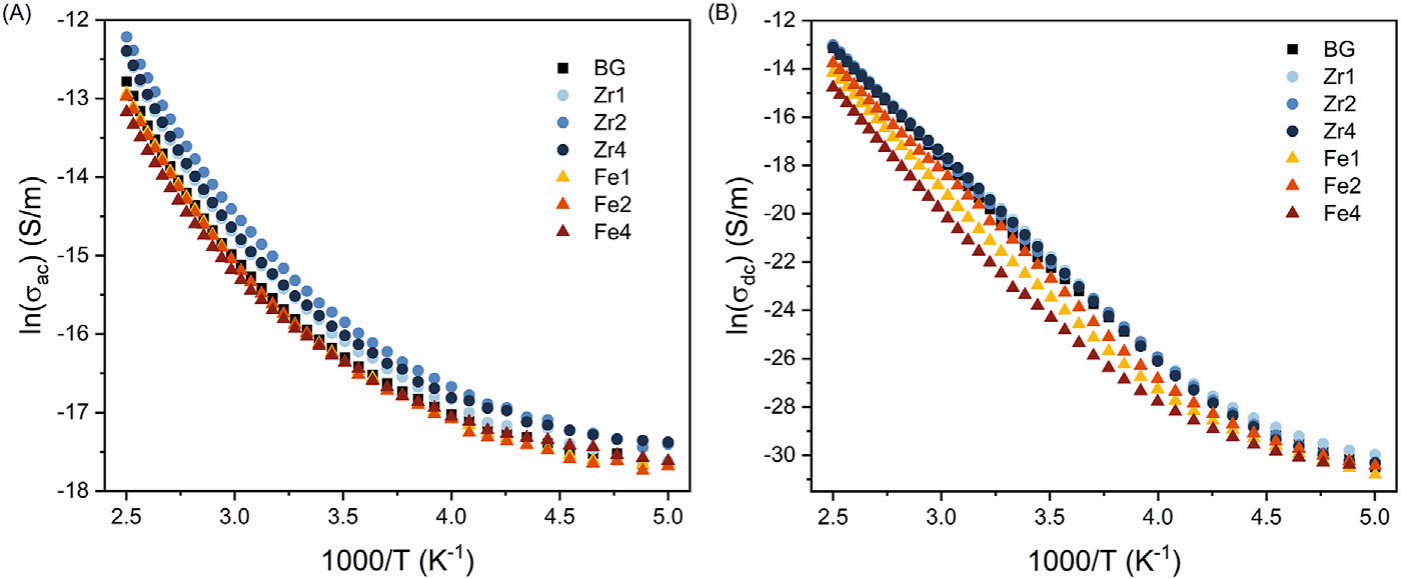
**(A)** AC conductivity at 10 kHz and **(B)** DC conductivity versus 1,000/T for the bioglass samples.

Figures 7A, B illustrate the temperature dependence of AC and DC conductivity on a logarithmic scale, respectively. As expected, conductivity increases with temperature due to enhanced charge carrier mobility. Above 270 K, the conductivity exhibits a linear relationship with temperature, enabling the determination of activation energy using the Arrhenius equation. Within this temperature range, ionic conductivity surpasses electronic conductivity, making ion transport the dominant conduction mechanism in these glasses. The conductivity of the bioglass is mainly attributed to the movement of network modifiers ions (such as Na^+^ and Ca^2+^) through the glass network (Obata et al., 2003; Keshri et al., 2021; Gavinho et al., 2024).

Table 2 reveals that the activation energy for DC conductivity exceeds that of AC conductivity. This disparity can be explained by the distinct nature of ion movement in each conduction type. DC conductivity necessitates long-range charge migration, whereas AC conductivity involves shorter, localized ion displacements. Therefore, DC conduction encounters higher energy barriers, resulting in a higher activation energy requirement (Kawamura et al., 2007; Hammami et al., 2022). Additionally, as shown in Table 2 and Figure 7, the incorporation of ZrO_2_ into the bioglass enhances both AC and DC conductivity, while the addition of Fe_3_O_4_ results in a decrease in both AC and DC conductivity. This can be attributed to the fact that ZrO_2_ insertion reduces the glass network’s rigidity, increasing the number of NBOs and consequently enhancing the mobility of modifier ions such as Na^+^ and Ca^2+^. Conversely, the addition of Fe_3_O_4_, especially at high concentrations, strengthens the glass structure by forming new cross-linking bonds with silica tetrahedra and therefore reducing ions mobility.

Overall, the structural transformations and changes in network connectivity (observed by XRD and Raman analysis) induced by oxide insertions played a crucial role in the observed electrical properties by influencing the mobility of network modifier ions (Na^+^ and Ca^2+^). In depolymerized glass networks with higher NBO content, these ions exhibit enhanced mobility, which improves the glass’s electrical properties. The bioglass modified with 2 mol% ZrO_2_, which exhibited a less rigid structure due to increased NBOs content (Figure 3), showed the highest dielectric constant and conductivity (Table 2). In contrast, at 4 mol% ZrO_2_, the formation of crystalline Na_4_Zr_2_(SiO_4_)_3_ phase (Figure 1A) reduced the number of NBO, increasing the network rigidity, and consequently decreasing these properties. For Fe_3_O_4_-modified bioglasses, higher Fe_3_O_4_ concentrations increased the dielectric constant due to the formation of Fe^3+^-O-Si bonds and Fe-related units (as shown by Raman analysis - Figure 2), which enhance polarizability. However, the conductivity decreased with increasing Fe_3_O_4_ concentration, as the formation of Fe^3+^ cross-linking bonds with silica tetrahedra increases glass rigidity and reduces ion mobility. Comparatively, ZrO_2_-modified glasses exhibited a greater ability to reduce network rigidity and increase NBOs content (Figure 3), thereby enhancing ion mobility and electrical conductivity.

The results of the electrical study give insights on the impact of oxide insertion on the charge storage capabilities of bioglass. A higher dielectric constant enables greater polarization and thus increased charge storage. The insertion of ZrO_2_ and Fe_3_O_4_ had a modest impact on the dielectric constant, with the 2 mol% ZrO_2_ sample exhibiting the highest value. Moreover, conductivity proved to be a significant factor affecting charge trapping within the glass matrix. Bioglasses containing ZrO_2_ demonstrated increased conductivity and enhanced ion mobility, which supported more efficient charge trapping. This indicates that the charge storage capability of these samples relies not only on their polarizability (as indicated by the dielectric constant) but also on the effectiveness of charge migration and trapping mechanisms within the glass structure.

### 3.3 Cytotoxicity

**FIGURE 8:**
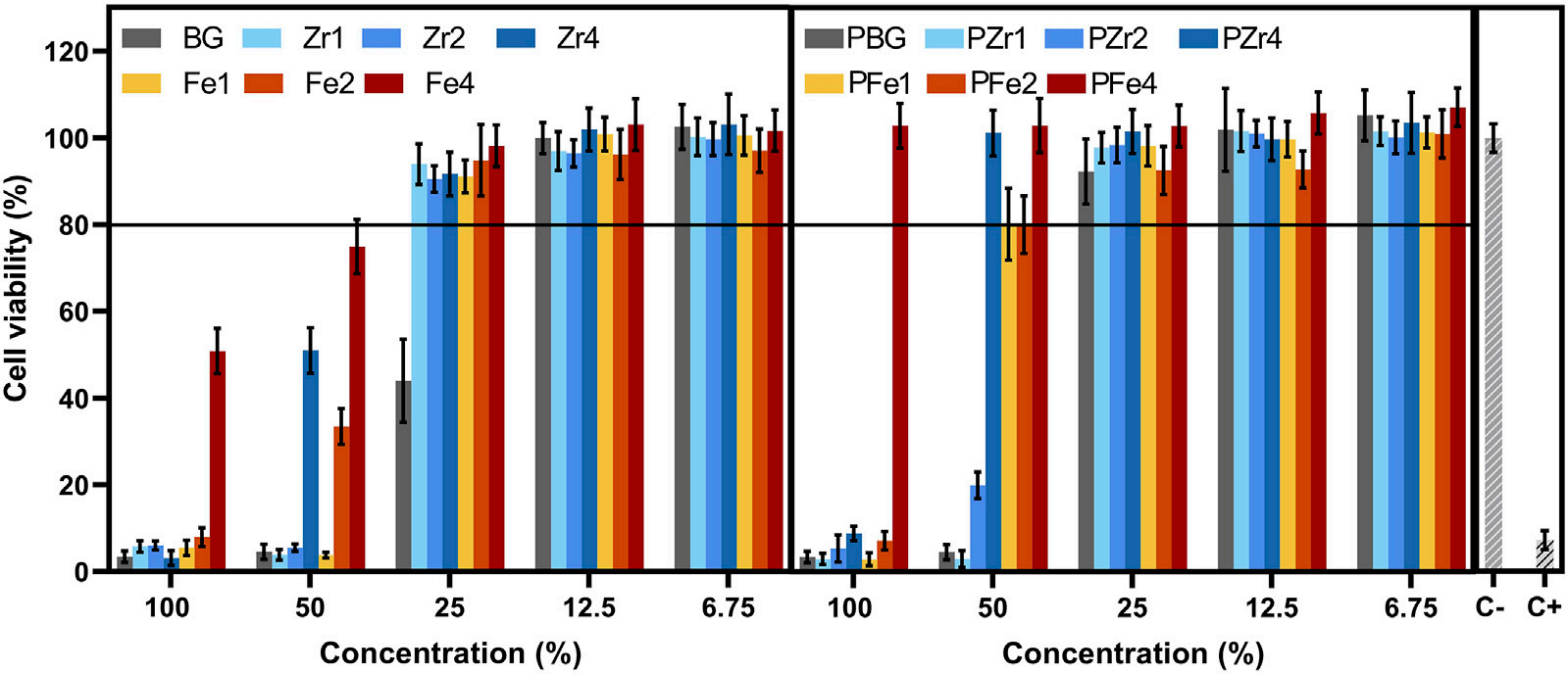
Cell viability of osteosarcoma cell line (Saos-2) after incubation with non-passivated (on the right) and passivated (on the left) bioglass extracts.

To assess the biocompatibility of the prepared bioglasses for potential biomedical applications, the viability of Saos-2 cells was evaluated after exposure to bioglass extracts. A resazurin assay was used to determine cell viability. As shown in Figure 8, the cytotoxicity of BG was significantly influenced by the type of metal oxide inserted into the bioglass network and the concentration of the extract. Non-passivated bioglass extracts containing ZrO_2_, which were not preconditioned with McCoy’s culture medium, exhibited significant cytotoxicity, reducing cell viability to less than 10% at a 100% extract concentration. In contrast, BG extracts with high Fe_3_O_4_ concentration demonstrated improved cell viability, even at a 100% extract concentration. While cytotoxic effects were still observed at higher concentrations, the findings suggest that Fe_3_O_4_ has a less pronounced cytotoxic effect than ZrO_2_. All the bioglasses, except the 45S5 bioglass, demonstrated cell viability above 70% at a concentration of 25%, indicating their non-toxic effect on Saos-2 cells. This suggests that the insertion of ZrO_2_, and Fe_3_O_4_ into BG can enhance the material’s biocompatibility. These results are consistent with previous research (Mondal et al., 2013; Moghanian et al., 2020; Ezealigo et al., 2021). As illustrated in Figure 8, the passivation process effectively mitigated the cytotoxicity of the extracts. The cytotoxicity of bioglass is related to an increase in local pH resulting from ion-exchange reactions when the sample is exposed to a cell culture medium during the initial 24 h period (El-Rashidy et al., 2017). During this interaction, bioglass undergoes degradation of its Si-O-Si bonds, releasing soluble silica in the form of Si(OH)_4_. This accelerates the dissolution rate and rises the pH of the surrounding environment, which can adversely impact cellular metabolism and function. However, it mitigates these alkalinization effects by establishing conditions that more closely resemble the *in vivo* environment, where living organisms actively regulate and maintain pH balance. The results obtained from the passivation process reveal that Fe_3_O_4_-containing bioglass exhibits excellent biocompatibility. Specifically, bioglass containing 4 mol% Fe_3_O_4_ demonstrated high cell viability even at a 100% extract concentration. Furthermore, at a 50% extract concentration, all Fe_3_O_4_-modified BGs exhibited no cytotoxic effects on Saos-2 cells while for the ZrO_2_-modified glasses, only the Zr4 does not show toxicity at this concentration. However, at 25% extract concentration all samples are no longer cytotoxic.

### 3.4 Antibacterial activity

**FIGURE 9:**
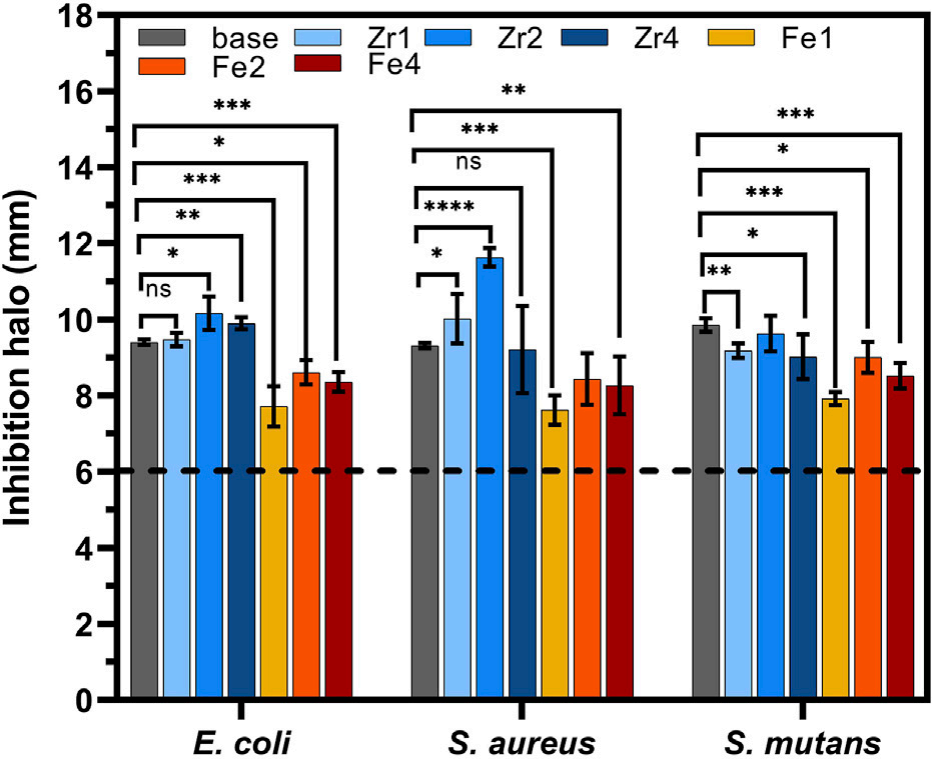
Measurement of the inhibition halo diameters of all samples against *E. coli*, *S. aureus*, and *S. mutans* bacteria after incubation for 24 h (statistical analysis was performed using unpaired t-test and the *p*-values indicate the statistical significance; ns: non-significant; *p ≤ 0.05; **p ≤ 0.01; ***p ≤ 0.001; ****p ≤ 0.0001).

Figure 9 illustrates the antibacterial properties of the samples, assessed using the agar disc diffusion method. All samples exhibited antibacterial activity, evidenced by the formation of inhibition zones surrounding the pellets. The average diameter of these zones exceeded 6 mm, the size of the pellets. Both the pH change toward alkalinity and the osmotic pressure generated by releasing ions like Na^+^ and Ca2^+^ into the surrounding media are major processes by which 45S5 BG inhibits bacterial growth (Hu et al., 2009; Drago et al., 2018). Additionally, incorporating oxides into the glass network can affect its antibacterial efficacy. An enhancement in the antibacterial properties of the bioglass was observed with the addition of ZrO_2_. Among the samples, Zr2 exhibited the highest antimicrobial activity, with inhibition halos measuring 10.17 mm, 11.63 mm, and 9.63 mm against *E. coli*, *S. aureus*, and *S. mutans*, respectively. The improved antibacterial activity in the glass with 2 mol% ZrO_2_ can be explained by the structural changes induced within the glass network. Through the fitting and deconvolution of Raman spectra (Figure 2), this sample showed the highest NBO content (Figure 3), suggesting a decrease in glass network connectivity. This reduced network connectivity due to the high concentration NBOs, facilitates the release of alkali metal ions (Na^+^, and Ca^2+^), leading to an increase in local pH, which promotes bacterial death. At higher ZrO_2_ concentrations, the antibacterial activity decreased, which can be attributed to the formation of crystalline phases that strengthen the glass network, as confirmed by XRD analysis (Figure 1A). The addition of Fe_3_O_4_ to the base material resulted in a notable decrease in antibacterial effectiveness. This reduction in antimicrobial activity can be explained by the presence of iron, which polymerizes the glass structure by forming new cross-links with silica tetrahedra, consequently hindering its dissolution rate. The sample containing 2 mol% Fe_3_O_4_ demonstrated the highest antibacterial activity, with average inhibition halo diameters of 8.61 mm, 8.44 mm, and 9.01 mm against *E. coli*, *S. aureus*, and *S. mutans*, respectively.

### 3.5 Bioactivity evaluation

**FIGURE 10:**
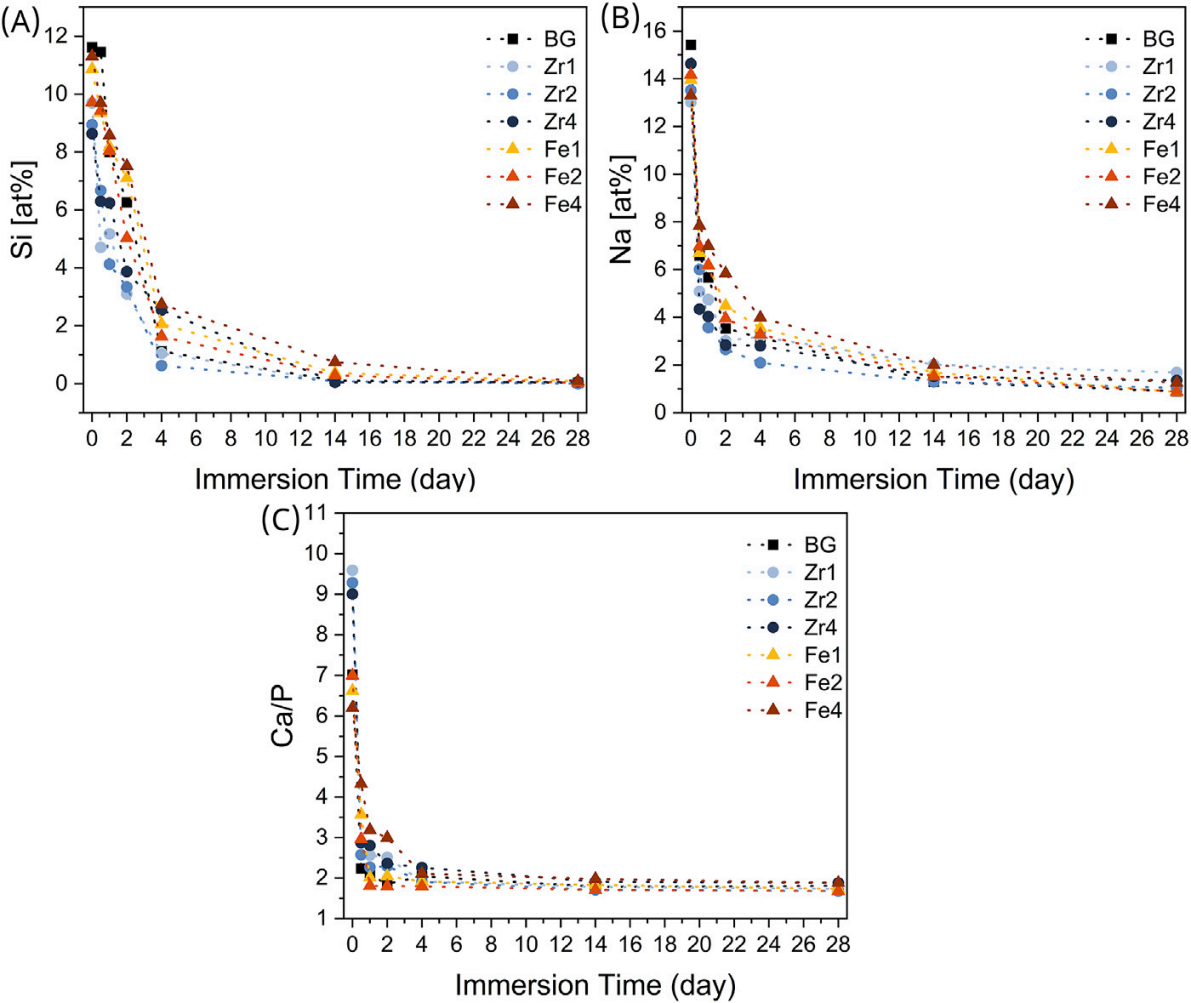
Variation of the atomic percentage of **(A)** silicon, **(B)** sodium ions, and **(C)** the ratio between calcium and phosphorous, presented on the surface of the bioglasses after SBF immersion.

Figures 10A–C illustrate the changes in the atomic percentages of Si, Na, and the Ca/P ratio, respectively, as a function of immersion time on the surfaces of various bioglass samples. A substantial reduction in Si and Na concentrations was observed on the sample surfaces during the initial period, followed by stabilization in subsequent days. This behaviour is attributed to the dissolution of these elements into the surrounding medium, coupled with the formation of a Ca-P-rich layer. Indeed, when bioglass comes into contact with SBF, an immediate exchange occurs between the monovalent (Na^+^) and divalent (Ca^2+^) ions present in the glass and the H^+^ ions in the fluid. The decrease in the amount of Na^+^ on the glass surface is evident in Figure 10B. At the initial immersion time, for samples containing ZrO_2_, this release is more pronounced, indicating higher glass reactivity. Compared to Fe_3_O_4_, the incorporation of ZrO_2_ into the bioglass network facilitates network expansion and consequently increases the ionic dissolution rate. The formation of a silica gel layer on the glass surface enhances the diffusion of Ca^2+^ and PO_4_^3+^ ions from the glass and the absorption of Ca and P ions from the solution, resulting in the formation of an amorphous calcium-phosphate layer. This series of reactions is illustrated in Figure 10A, C by the decrease in silicon and the decreasing Ca/P ratio, converging towards a value of 1.67, characteristic of hydroxyapatite (Beaufils et al., 2019; Boukha et al., 2019).

**FIGURE 11:**
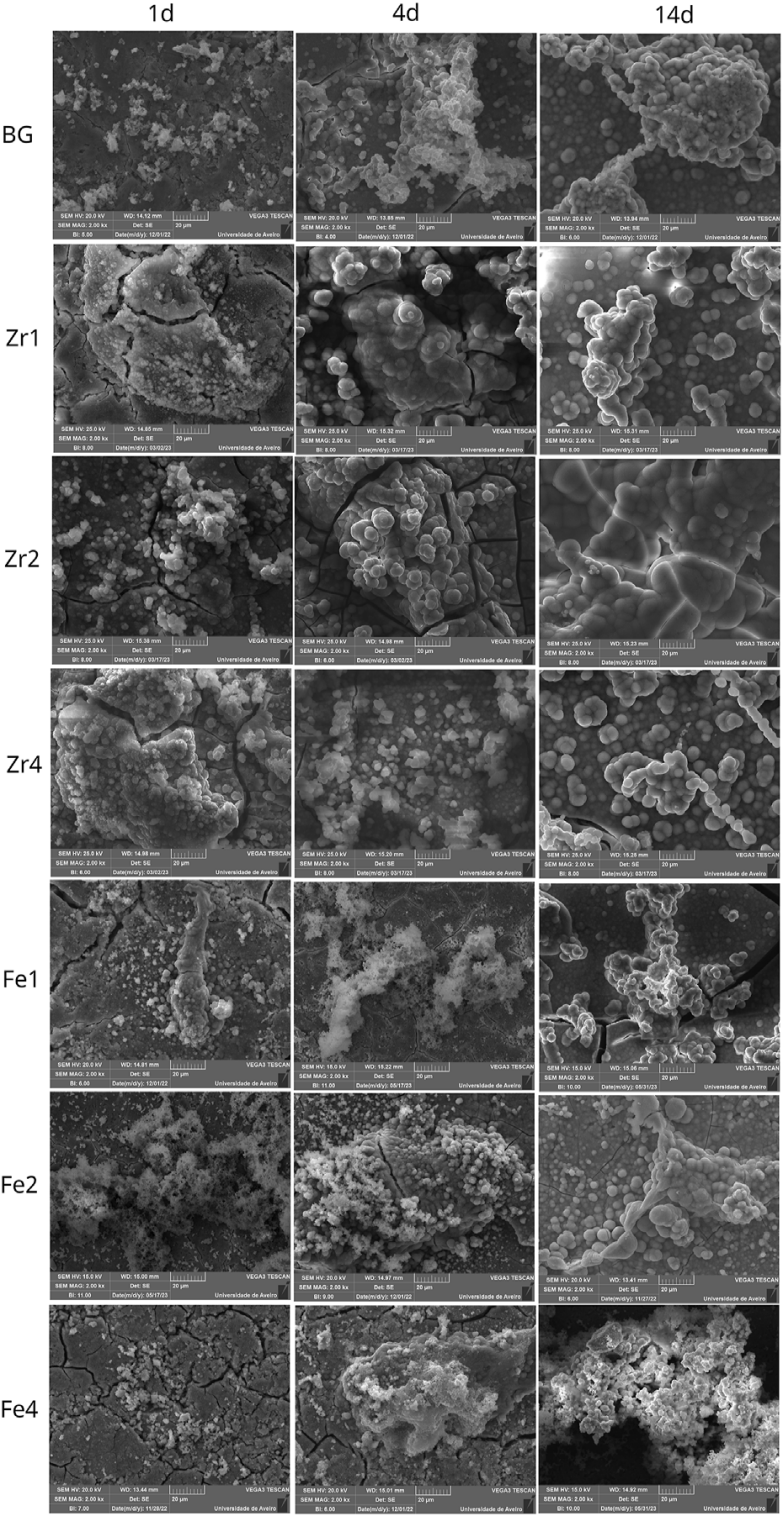
SEM micrographs of different bioglass surfaces after immersion in SBF for 1 d, 4 d, and 14 d.

SEM micrographs of the surface of different bioglass samples after 0, 1, 4, and 14 d of immersion in SBF are shown in Figure 11. The formation of an apatitic layer on the surface was confirmed through SEM, revealing spherical particles with cauliflower-like morphologies, indicative of the bioactivity of the BGs. As immersion time increased, these apatite particles aggregated and became denser, eventually covering the entire surface after 14 d. These observations provide strong evidence of the osteoconductive potential of the prepared samples, demonstrating their ability to promote bone growth and regeneration. From Figure 11, it can be observed that the size and amount of spherical apatite particles formed on the bioglass surface, particularly during the initial days of SBF immersion, vary depending on the type and concentration of oxides inserted into the glass network. This variation in bioactivity is influenced by the structural changes induced by oxide insertion. Specifically, the presence of a depolymerized glass network facilitates ion exchange between the bioglass and SBF upon immersion, thereby enhancing bioactivity. The bioglass containing 2 mol% ZrO_2_ exhibited a higher NBO content (Figure 3) which increased the mobility and release of network modifier ions (Na^+^ and Ca^2+^). This led to a rise in the glass dissolution rate during SBF immersion. Consequently, the sample containing 2 mol% ZrO_2_ exhibited larger apatite particles during the initial days of SBF immersion, indicating enhanced bioactivity. However, when the ZrO_2_ content exceeded 2 mol%, smaller apatite particles were observed during the early stages of immersion. This is likely due to the formation of crystalline phases, as confirmed by the XRD analysis, which increased glass rigidity and consequently reduced the dissolution rate and bioactivity. In contrast, bioglasses containing Fe_3_O_4_ demonstrated lower bioactivity compared to those with ZrO_2_ and the 45S5 BG during the initial days of SBF immersion. This is evident in the SEM images, which show smaller apatite particles. This reduced bioactivity may be attributed to the stronger connectivity of the glass network, which limits ion release and consequently diminishes bioactivity.

**FIGURE 12:**
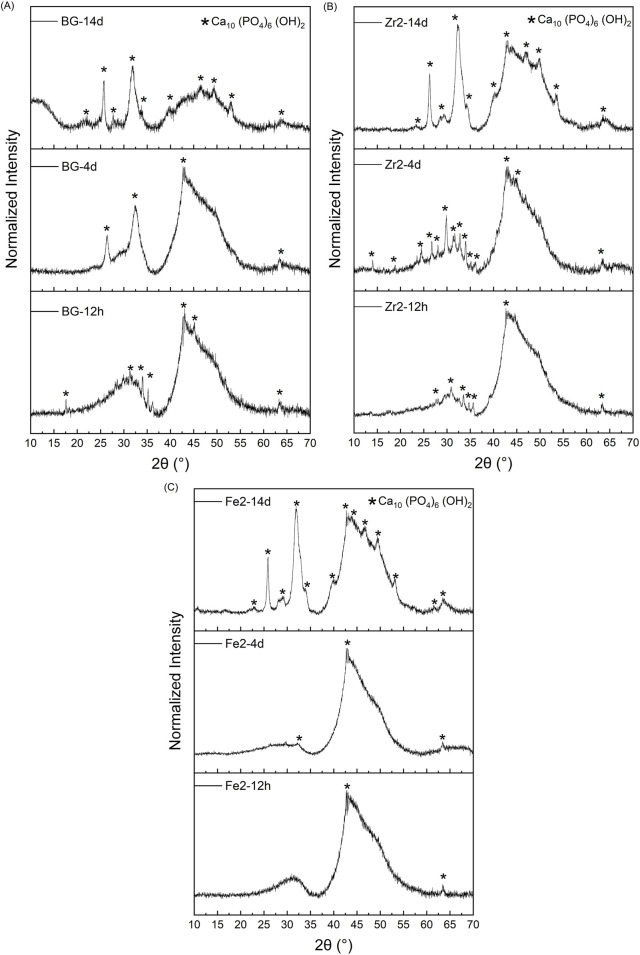
XRD patterns for **(A)** BG, **(B)** Zr2, and **(C)** Fe2 samples after SBF immersion for 12 h, 4 d, and 14 d.

Figures 12A–C show the XRD patterns of the 45S5 BG, Zr2, and Fe2 samples, respectively, after immersion in SBF for 12 h, 4 d, and 14 d to evaluate whether the Ca-P layer formed on the surface is amorphous or crystalline. Prior to SBF immersion, the XRD patterns (Figure 1A) displayed no distinct diffraction peaks, only a broad amorphous hump. After 12 h of immersion in SBF, a crystalline phase began to form, with a diffraction peak corresponding to hydroxyapatite, Ca_10_(PO_4_)_6_(OH)_2_ (ICCD No. 00-001–1,008) (Miola et al., 2015). As immersion time progressed, the crystallinity of the samples increased due to the growth of the hydroxyapatite layer on the surface. During the initial stages of immersion, 45S5 BG and Zr2 exhibited higher crystallinity compared to the Fe2 sample, indicating greater bioactivity. However, with prolonged immersion, the hydroxyapatite layer on the Fe2 sample became comparable to that of the other samples. This suggests that while Fe initially reduces the bioactivity of the bioglass, it does not impede the long-term formation of hydroxyapatite. These findings are consistent with SEM-EDS results, confirming the bioactivity of the bioglasses.

## 4 Conclusion

In this work, a detailed investigation into the structural, electrical, and biological properties of bioactive glasses modified with ZrO_2_, and Fe_3_O_4_ was conducted to evaluate their potential application as implant coatings. Our findings suggest that the introduction of these oxides led to changes in the glass network structure and connectivity. For the bioglass modified with ZrO_2_, structural changes were identified through XRD analysis, with a high ZrO_2_ content sample showing the emergence of crystalline phase. In the case of glasses modified with Fe_3_O_4_, Raman analysis detected the appearance of new vibrational modes associated with Fe-related structural units. The insertion of these oxides and the resulting structural changes significantly affected the bioglass connectivity, which in turn influenced its electrical and biological properties. Compared to Fe_3_O_4_, the insertion of the ZrO_2_ into the bioglass network enhanced the conductivity and ion mobility. This improvement is attributed to the variation in the amount of non-bridging oxygen ions (NBOs), which was higher in glasses containing ZrO_2_. These structural changes also influenced the antibacterial activity and bioactivity of the bioglasses. The samples modified with ZrO_2_ demonstrated superior antibacterial activity and bioactivity compared to the base bioglass and the bioglass modified with Fe_3_O_4_. In contrast, the incorporation of Fe_3_O_4_ resulted in a reduction in both bioactivity and antibacterial activity. This decrease can be attributed to the effect of iron, which increases the rigidity of the glass network, thereby limiting ion release and reducing its bioactive and antibacterial performance. Among all the samples, the bioglass with 2mol% ZrO_2_ showed the best antibacterial and bioactivity, suggesting that it is the most suitable for implant coating. The enhanced bioactivity observed in these glasses, particularly in the bioglass with 2 mol% ZrO_2_, indicates their potential to promote osseointegration by facilitating the rapid formation of an apatite layer. This layer is critical for bonding implants to bone tissue and improving implant stability. Enhanced stability can reduce micromovements, thereby lowering the risk of bacterial infection. Furthermore, the superior antibacterial properties of these glasses suggest they could play a significant role in minimizing implant-associated infections, which remain a major challenge in clinical implantology. Moving forward, further studies focusing on *in vivo* testing are essential to validate the long-term stability and performance of these materials under physiological conditions. Such investigations are necessary to translate these findings into clinical practice and address existing challenges in implantology.

## Data Availability

The raw data supporting the conclusions of this article will be made available by the authors, without undue reservation.
